# Impact of *EGFR* Mutation Detection Methods on the Efficacy of Erlotinib in Patients with Advanced *EGFR*-Wild Type Lung Adenocarcinoma

**DOI:** 10.1371/journal.pone.0107160

**Published:** 2014-09-12

**Authors:** Jeng-Sen Tseng, Chih-Liang Wang, Ming-Shyan Huang, Chung-Yu Chen, Cheng-Yu Chang, Tsung-Ying Yang, Chi-Ren Tsai, Kun-Chieh Chen, Kuo-Hsuan Hsu, Meen-Hsin Tsai, Sung-Liang Yu, Kang-Yi Su, Chih-Wei Wu, Cheng-Ta Yang, Yuh-Min Chen, Gee-Chen Chang

**Affiliations:** 1 Division of Chest Medicine, Department of Internal Medicine, Taichung Veterans General Hospital, Taichung, Taiwan; 2 Institute of Biomedical Sciences, National Chung Hsing University, Taichung, Taiwan; 3 Department of Thoracic Medicine, Chang Gung Memorial Hospital, Taoyuan, Taiwan; 4 Department of Medicine, Chang Gung University, Taoyuan, Taiwan; 5 Division of Pulmonary and Critical Care Medicine, Department of Internal Medicine, Kaohsiung Medical University Hospital, Kaohsiung, Taiwan; 6 Faculty of Medicine, College of Medicine, Kaohsiung Medical University, Kaohsiung, Taiwan; 7 Department of Internal Medicine, National Taiwan University Hospital Yunlin Branch, Yunlin County, Taiwan; 8 Division of Chest Medicine, Department of Internal Medicine, Far Eastern Memorial Hospital, Taipei, Taiwan; 9 Faculty of Medicine, School of Medicine, National Yang-Ming University, Taipei, Taiwan; 10 Department of Pediatrics, Taichung Veterans General Hospital, Taichung, Taiwan; 11 Institute of Molecular Biology, National Chung-Hsing University, Taichung, Taiwan; 12 Division of Critical Care and Respiratory Therapy, Department of Internal Medicine, Taichung Veterans General Hospital, Taichung, Taiwan; 13 Institute of Statistical Science, Academia Sinica, Taipei, Taiwan; 14 Department of Clinical Laboratory Sciences and Medical Biotechnology, College of Medicine, National Taiwan University, Taipei, Taiwan; 15 Center for Optoelectronic Biomedicine, College of Medicine, National Taiwan University, Taipei, Taiwan; 16 Graduate Institute of Pathology, College of Medicine, National Taiwan University, Taipei, Taiwan; 17 Department of Laboratory Medicine, National Taiwan University Hospital, Taipei, Taiwan; 18 Center of Genomic Medicine, National Taiwan University, Taipei, Taiwan; 19 Department of Chest Medicine, Taipei Veterans General Hospital, Taipei, Taiwan; 20 Department of Respiratory Therapy, College of Medicine, Chang Gung University, Taoyuan, Taiwan; 21 College of Medical Science and Technology, Taipei Medical University, Taipei, Taiwan; 22 Comprehensive Cancer Center, Taichung Veterans General Hospital, Taichung, Taiwan; Seoul National University, Republic of Korea

## Abstract

**Introduction:**

Methods used for *epidermal growth factor receptor (EGFR)* mutation testing vary widely. The impact of detection methods on the rates of response to EGFR-tyrosine kinase inhibitors (TKIs) in *EGFR*-wild type (wt) lung adenocarcinoma patients is unknown.

**Methods:**

We recruited the Group-I patients to evaluate the efficacy of erlotinib in patients with *EGFR*-wt lung adenocarcinoma by either direct sequencing (DS) or mutant type-specific sensitive (MtS) methods in six medical centers in Taiwan. Cross recheck of *EGFR* mutations was performed in patients who achieved objective response to erlotinib and had adequate specimens. The independent Group-II lung adenocarcinoma patients whose *EGFR* mutation status determined by DS were recruited to evaluate the potential limitations of three MtS methods.

**Results:**

In Group-I analysis, 38 of 261 *EGFR*-wt patients (14.6%) achieved partial response to erlotinib treatment. Nineteen patients (50.0%) had adequate specimens for cross recheck of *EGFR* mutations and 10 of them (52.6%) had changes in *EGFR* mutation status, 5 in 10 by DS and 5 in 9 by MtS methods originally. In Group-II analysis, 598 of 996 lung adenocarcinoma patients (60.0%) had detectable *EGFR* mutations. The accuracy rates of the three MtS methods, MALDI-TOF MS, Scorpions ARMS and Cobas, were 87.8%, 86.8% and 85.8%, respectively.

**Conclusions:**

A significant portion of the erlotinib responses in *EGFR*-wt lung adenocarcinoma patients were related to the limitations of detection methods, not only DS but also MtS methods with similar percentages. Prospective studies are needed to define the proper strategy for *EGFR* mutation testing.

## Introduction

In recent years, epidermal growth factor receptor (EGFR)-targeted therapy has emerged as a novel and effective strategy in lung cancer management with major benefits in patients with *EGFR* activating mutations. Not only in front line but also in subsequent therapy, EGFR-tyrosine kinase inhibitors (TKIs) in comparison with chemotherapy have demonstrated significantly higher response rate and longer progression-free survival (PFS) in patients with *EGFR*-mutant non-small cell lung cancer (NSCLC) [1]. Moreover, EGFR-TKIs therapy is associated with a better quality of life [2]–[4]. Therefore, many studies suggested EGFR-TKI as the first line therapy for *EGFR*-mutant NSCLC patients [5], [6].

Despite the close association between *EGFR* mutations and EGFR-TKIs responsiveness, NSCLC patients, who had no detectable *EGFR* mutations, have been reported to benefit from the EGFR-TKIs [7]–[9] and erlotinib remains an important second-line treatment option in the clinical practice guidelines for NSCLC, irrespective of biological characteristics [10]–[12]. A pooled analysis, which included three Phase III randomized controlled trials that compared the efficacy of erlotinib with other therapies in *EGFR*-wild type (*EGFR*-wt) NSCLC patients, also suggested a significant benefit of erlotinib treatment [13]. However, various *EGFR* mutation detection methods were used in studies regarding the efficacy of erlotinib in *EGFR*-wt NSCLC and their false negative rates have been suspected to be a possible reason for the responses to EGFR-TKIs in patients without detectable *EGFR* mutations [14], [15].

Direct sequencing (DS) can detect all existing mutations but is limited by its lower sensitivity [16]. Mutant type-specific sensitive (MtS) methods, such as the protein nucleic acid-locked nucleic acid polymerase chain reaction (PNA-LNA PCR) clamp or Scorpions amplification refractory mutation system (ARMS) can detect specific and known mutations but not rare mutations [17]. The results of studies that investigated the association between discrepant *EGFR* mutation results by different methods and the outcomes of EGFR-TKIs treatment were inconsistent [18], [19]. The impact of detection methods on the rates of response to EGFR-TKIs in *EGFR*-wt lung adenocarcinoma patients is unknown. We conducted this study to evaluate the impact of detection methods on the efficacy of erlotinib in patients with advanced *EGFR*-wt lung adenocarcinoma.

## Materials and Methods

### Patients

We recruited two independent groups of patients for participation in this study. From August 2005 to March 2013, we evaluated the efficacy of erlotinib in lung adenocarcinoma patients (Group –I) with *EGFR*-wt status assessed by regular methods (either DS or MtS methods) used in six participating medical centers in Taiwan (Taichung Veterans General Hospital, (TCVGH) Taipei Veterans General Hospital, Chang Gung Memorial Hospital (CGMH), Kaohsiung Medical University Hospital, National Taiwan University Hospital Yunlin Branch and Far Eastern Memorial Hospital). Inclusion criteria for Group-I patients were advanced lung adenocarcinoma without detectable *EGFR* mutations (exon 18, 19, 20 and 21) at initial molecular analysis, a history of erlotinib treatment for more than 7 days and clinically measurable disease. Patients were excluded if they had other active malignancy, incomplete data records or received other treatments concurrently. All patients received erlotinib at a daily dose of 150 mg initially. TNM (tumor, node, and metastases) staging was done according to the 7th edition of the American Joint Committee for Cancer (AJCC) staging system [20].

From January 2000 to June 2013 we evaluated consecutive lung adenocarcinoma patients of any stage who were treated in TCVGH and CGMH (Group-II). We assessed their *EGFR* mutation status by DS and calculated the number of *EGFR* mutations that would not be detected by three MtS methods. This study was approved by the institutional review boards of the participating institutions, including Institutional Review Board of Taichung Veterans General Hospital, Institutional Review Board of Taipei Veterans General Hospital, Chang Gung Medical Foundation Institutional Review Board, Kaohsiung Medical University Chung-Ho Memorial Hospital Institutional Review Board, National Taiwan University Hospital Research Ethics Committee and Far Eastern Memorial Hospital Research Ethics Review Committee. Written informed consent for genetic testing and clinical data records was obtained from all patients.

### Data records and response evaluation

Clinical data for analysis included age, gender, Eastern Cooperative Oncology Group performance status (ECOG PS), tumor stage, prior chemotherapies, smoking status, *EGFR* detection methods and erlotinib treatment history. The adverse events associated with erlotinib treatment including interstitial lung disease and grade 3–4 hepatotoxicity were recorded. Chest computed tomographies, including the liver and adrenal glands, and other required imaging studies for response evaluation were reviewed by two chest physicians. Unidimensional measurements as defined by Response Evaluation Criteria in Solid Tumors version 1.1 were used in this study [21]. The objective response rate (ORR), disease control rate (DCR), PFS and overall survival (OS) of erlotinib treatment were assessed.

### EGFR mutation tests

For the Group-I patients, several molecular tests, including DS, PNA-LNA PCR clamp, Scorpions ARMS (EGFR RGQ PCR Kit) and matrix-assisted laser desorption ionization-time of flight mass spectrometry (MALDI-TOF MS) were used for *EGFR* mutation analysis [9], [22]–[24], which depended on the laboratory facilities of participating institutions. As for DS, PNA-LNA PCR clamp and MALDI-TOF MS methods, DNA was extracted from the tumors for *EGFR* mutation analysis as previously described [9], [24] and the detection spectrum of PNA-LNA PCR clamp and MALDI-TOF MS is summarized in Table S1. As for Scorpions ARMS, commercialized kit was used and samples were processed according to the manufacturer's protocol [25]. We defined PNA-LNA PCR clamp, Scorpions ARMS and MALDI-TOF MS as the MtS methods to be compared with DS for evaluation of the influence of detection methods on the efficacy of erlotinib treatment. For the Group-II patients, we assessed their *EGFR* mutation status by DS and calculated the number of *EGFR* mutations that would not be detected by MALDI-TOF MS and two other commercialized methods, Scorpions ARMS [25] and Cobas *EGFR* Mutation Test [26].

### Statistical methods

Univariate analysis of ORR and DCR were performed using Fisher's exact test to evaluate the effects of clinical factors relating to patients' characteristics and *EGFR* detection methods. Multivariate analyses of ORR and DCR were performed using logistic regression model. The Kaplan–Meier method was used to estimate PFS and OS. Differences in survival time in regard to *EGFR* detection methods were analyzed using the log-rank test. Multivariate analyses of PFS and OS were performed using Cox proportional hazard model. All statistical tests were done with SPSS 15.0 (SPSS Inc., Chicago, IL, USA). Two-tailed tests and p values <0.05 for significance were used.

## Results

### Efficacy and adverse effects of erlotinib in lung adenocarcinoma patients without detectable EGFR mutations at initial molecular testing

A total of 261 patients were included in Group-I and the baseline characteristics are shown in Table S2. The median age was 62 years, 162 patients (62.1%) were male, 138 patients (52.9%) were non-smokers and 174 patients (66.7%) had ECOS PS 0–1. Initial *EGFR* mutation status was assessed by DS in 191 patients (73.2%) and by MtS methods in 70 patients (26.8%).

Thirty-eight patients achieved partial response (PR) and 52 had stable disease. No patient achieved complete response. The ORR and DCR were 14.6% and 34.5%, respectively. The responses and survival analysis are summarized in Table 1. The median PFS and OS were 1.9 (95% CI 1.7–2.1) and 8.3 (95% CI 5.9–10.7) months respectively. The 1-year survival rate was 25.7%. PFS and OS were significantly longer in patients with disease control than in those with progressive disease (both P<0.001).

**Table 1:** Efficacy of erlotinib in 261 lung adenocarcinoma patients without detectable *EGFR* mutations at initial molecular testing.

Best Response	
	Patient No. (%)
Complete response (CR)	0 (0)
Partial response (PR)	38 (14.6)
Objective response rate	38 (14.6)
(ORR = CR + PR)	
Stable disease (SD)	52 (19.9)
Disease control rate	90 (34.5)
(DCR = CR + PR + SD)	
Progressive disease (PD)	171 (65.5)

Survival			
	Median PFS (m)^a^ (95% CI)	Median OS (m)^b^ (95% CI)	1-year survival rate (%)^b^
PR	11.0 (8.1–13.9)	32.6 (26.5–38.7)	-
SD	5.8 (3.4–8.2)	18.4 (7.4–29.3)	-
DC	8.4 (7.2–9.6)	30.0 (15.9–44.0)	-
PD	1.4 (1.2–1.6)	4.9 (3.7–6.1)	-
Total	1.9 (1.7–2.1)	8.3 (5.9–10.7)	25.7

PFS, progression-free survival; OS, overall survival; PR, partial response; SD, stable disease; DC, disease control; PD, progressive disease.

a 38 patients are still under erlotinib treatment without PD.

b 102 patients are still alive.

Results of univariate analysis of ORR are shown in Table 2. There was no significant association between the erlotinib treatment responses and patients' age, gender, smoking status and ECOG PS. Furthermore, the ORR of patients whose *EGFR* mutation status was assessed by DS and by MtS methods were comparable (14.1 vs. 15.7%, P = 0.843). No covariate reached the significance level to enter the multivariate logistic regression model.

**Table 2:** Univariate analysis of objective response rate of erlotinib treatment in lung adenocarcinoma patients without detectable *EGFR* mutations at initial molecular testing.

	Patient No.	ORR (%)	P value
Gender			0.209
Male	162	12.3	
Female	99	18.2	
Age (yrs)			0.478
≤ 65	158	13.3	
> 65	103	16.5	
ECOG PS			0.358
0–1	174	16.1	
≧ 2	87	11.5	
Smoking			0.113
NS	138	18.1	
C/FS	123	10.6	
*EGFR* methods			0.843
Direct sequencing	191	14.1	
Sensitive methods^a^	70	15.7	

ORR, objective response rate; ECOG PS, Eastern Cooperative Oncology Group performance status; NS, nonsmoker; C/FS, current or former smoker; EGFR, epidermal growth factor receptor.

a Include Scorpions ARMS, MALDI-TOF MS and PNA-LNA PCR clamp methods.

**Figure 1:**
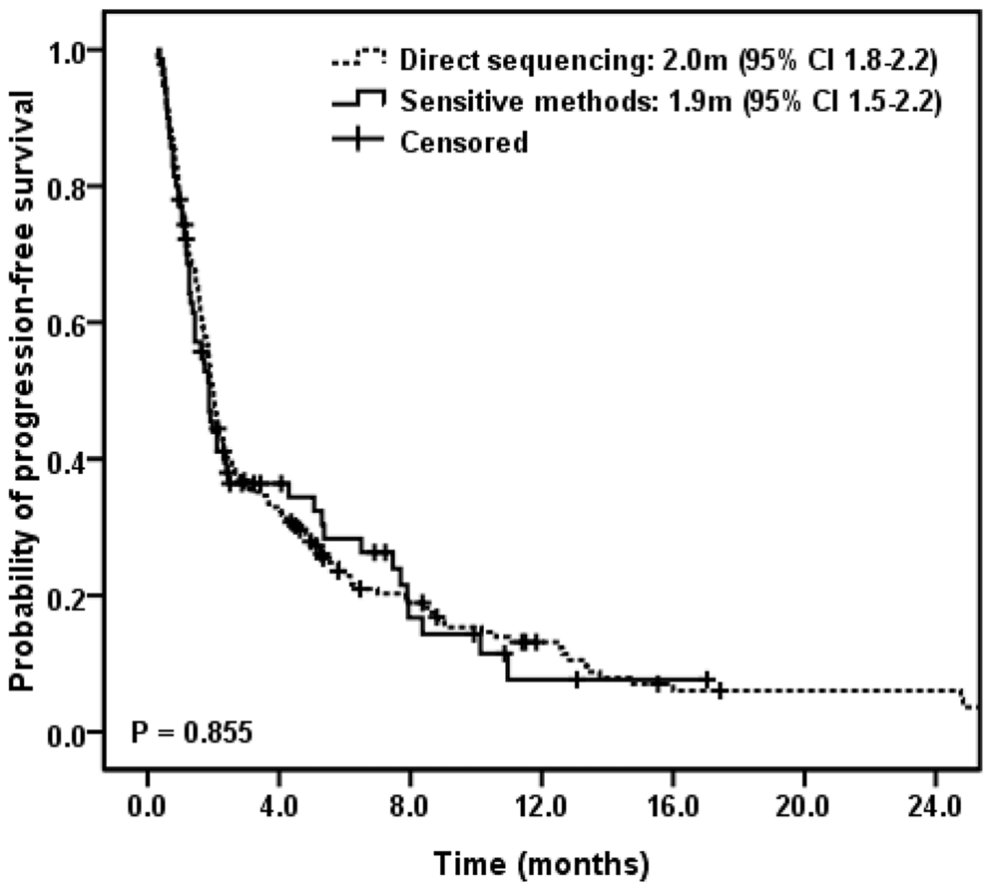
Kaplan-Meier plot showing progression-free survival according to different *EGFR* mutation detection methods.

Kaplan–Meier curve of PFS in regard to detection methods is shown in Figure 1. There was no significant difference in PFS between patients with *EGFR*-wt tumors assessed by DS and by MtS methods (2.0 vs. 1.9 months, P = 0.855) and similar survival periods were noted in OS analysis (8.3 vs. 10.9 months, P = 0.782). Patients' characteristics other than detection methods did not correlate significantly with PFS and OS (data not shown) and no covariates reached the significance level to enter the multivariate Cox proportional hazard model.

As for adverse events, interstitial lung disease occurred in 2 patients (0.8%) and 8 patients (3.1%) had grade 3–4 hepatotoxicity. None of these adverse events led to death.

### Cross recheck of EGFR mutation status in EGFR-wt patients with objective responses to erlotinib treatment

Thirty-eight of 261 patients (14.6%) in Group-I achieved objective responses to erlotinib treatment. Nineteen of them (50.0%) had adequate specimens for *EGFR* mutation status cross recheck. Initial molecular testing was performed by DS in 10 patients (52.6%) and by MtS methods in 9 patients (47.4%). Patients with *EGFR*-wt mutation status assessed by DS were rechecked by MtS methods and vice versa. In this study, the MtS method used for the recheck was MALDI-TOF MS.

Results of *EGFR* mutation status recheck are summarized in Table 3. Of 10 patients with *EGFR*-wt mutation status assessed by DS, 5 patients (50.0%) were found to have *EGFR* mutations by MALDI-TOF MS, including 2 with Del E746_A750, 1 with L858R and 2 with complex mutations, Del E746_A750/T790M and L858R/T790M. Of 9 patients with *EGFR*-wt mutation status assessed by MtS methods, 5 patients (55.6%) were found to have *EGFR* mutations by DS, including 4 with exon 19 deletions (Del L745_A750>R, Del K746_T751>VP, Del L747-A750>P and Del L747_T751>N) and 1 with I706T, a point mutation at exon 18. Of theses mutations, only Del L747_A750>P can be detected by available MtS methods. *EGFR* mutation status of patient S4 was assessed as wild type by PNA-LNA PCR clamp in September 2011. Our laboratory facility was not able to detect Del L747_A750>P until September 2013 when we added new mutation detection probes. In total, 10 of 19 patients (52.6%) had changes in *EGFR* mutation status.

**Table 3:** Results of *EGFR* mutation status recheck in part of patients with objective response to erlotinib treatment.

	Demographic data				Cross recheck of *EGFR* mutations		Efficacy of erlotinib	
Pt	Age	Gender	Smoking	ECOG	Method	Result	Prior C/T	PFS (m)
**Direct sequencing group**								
D1	82	M	CS	1	MS	Del E746_A750	1	8.6
D2	60	M	NS	1	MS	Del E746_A750	0	12.8
D3	61	M	FS	1	MS	Unfound	1	5.0
D4	73	F	NS	3	MS	Unfound	0	15.5^b^
D5	77	M	NS	1	MS	Unfound	1	11.0
D6	82	M	FS	2	MS	Unfound	1	2.9^b^
D7	84	M	NS	2	MS	Unfound	0	5.8^b^
D8	64	F	NS	2	MS	Del E746_A750/T790M	0	5.3^b^
D9	82	M	CS	2	MS	L858R	1	5.1^b^
D10	49	F	NS	1	MS	L858R/T790M	2	8.4
**Sensitive method group**								
S1	77	F	NS	1	DS	Del L747_T751>N	1	13.1^b^
S2	73	M	FS	1	DS	Del K745_A750>R	1	2.4^b^
S3	58	F	NS	2	DS	Del E746_T751>VP	1	5.1
S4	62	F	NS	3	DS	Del L747_A750>P^a^	3	6.5
S5	61	F	NS	1	DS	I706T	2	7.9
S6	65	F	NS	1	DS	Wild type	1	7.7
S7	77	M	CS	1	DS	Wild type	0	6.9
S8	65	M	NS	2	DS	Wild type	2	4.1^b^
S9	59	F	NS	1	DS	Wild type	1	10.1

EGFR, epidermal growth factor receptor; ECOG, Eastern Cooperative Oncology Group performance status; C/T, chemotherapy; PFS, progression-free survival; CS, current smoker; FS, former smoker; NS, non-smoker; MS, matrix-assisted laser desorption ionization-time of flight mass spectrometry; DS, direct sequencing.

a *EGFR* mutation status of patient S4 was analyzed as wild type by PNA-LNA PCR clamp in September 2011 and our facility was able to detect Del L747_A750>P since September 2013 by adding new mutation detection probes.

b Still under erlotinib treatment without progression.

### Using independent direct sequencing cohort to evaluate the limitations of three mutant type-specific sensitive methods

Results of the analysis of Group-I patients showed that both DS and MtS methods were unable to detect a significant portion of *EGFR*-mutations. As DS is well known by its low detection sensitivity, which may miss up to 20–25% *EGFR* mutations in comparison with varied MtS methods [16], [18], [19], we focused on how many *EGFR* mutations would not be detected by MtS methods. Therefore, we recruited the independent Group-II patients, whose *EGFR* mutation status was assessed by DS method, to evaluate the potential limitations of MtS methods.

In total, 996 consecutive lung adenocarcinoma patients were included in Group-II and the baseline characteristics are shown in Table S3. We used the database to evaluate the detectability of three MtS methods, including MALDI-TOF MS, which has been established at National Taiwan University Center of Genomic Medicine as one of our standard *EGFR* detection methods and two commercialized methods, Scorpions ARMS and Cobas *EGFR* Mutation Test.

**Figure 2:**
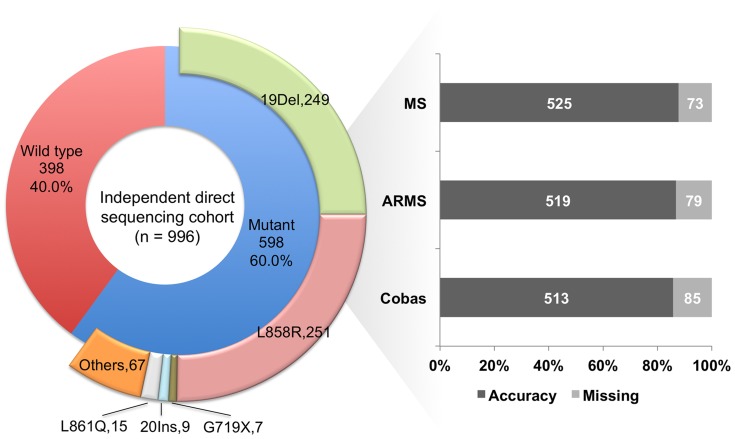
Detectability analysis of various mutant type-specific sensitive methods in an independent direct sequencing cohort (complex mutations were categorized into the group “others”; “missing” indicated partly detectable plus undetectable *EGFR* mutations; MS, matrix-assisted laser desorption ionization-time of flight mass spectrometry; ARMS, Scorpions amplification refractory mutation system).

Figure 2 shows that 598 of the 996 patients (60.0%) had detectable *EGFR* mutations. The exon 19 deletions (41.6%) and L858R (42.0%) were the major mutation types. Complex mutations of any combinations were categorized into the group “others”. In the detectability analysis, we defined fully and partly detectable as full spectrum of mutation(s) or only part of complex mutations could be detected respectively. Detection rate is the percentage of fully plus partly detectable mutations and the accuracy rate is the percentage of fully detectable mutations. As shown in Figure 2, MALDI-TOF MS, Scorpions ARMS and Cobas could not detect or only partly detected the mutation types in 73, 79 and 85 patients respectively. The detection rates of MALDI-TOF MS, Scorpions ARMS and Cobas were 92.8%, 92.8% and 91.8% and the accuracy rates of the three methods were be 87.8%, 86.8% and 85.8% respectively as disclosed in Table 4. Table S4 shows the full *EGFR* mutation spectrum of Group-II patients. It also indicated the detectability of three MtS methods and the mutations associated with disease control in response to EGFR-TKIs therapy according to treatment history at our facilities and the DNA-Mutation Inventory to Refine and Enhance Cancer Treatment (DIRECT) database [27].

**Table 4:** *EGFR* mutation detectability of various mutant type-specific sensitive methods in an independent cohort analyzed by direct sequencing (a total of 996 patients, of whom 598 harbored *EGFR* mutations).

	MS	ARMS	Cobas
Detectability, n			
Fully detectable^a^	525	519	513
Partly detectable^b^	30	36	36
Undetectable	43	43	49
Detection rate, (%)	92.8 (555/598)	92.8 (555/598)	91.8 (549/598)
(Fully + Partly detectable)			
Accuracy rate (%)	87.8 (525/598)	86.8 (519/598)	85.8 (513/598)
(Fully detectable)			

EGFR, epidermal growth factor receptor; MS, matrix-assisted laser desorption ionization-time of flight mass spectrometry; ARMS, Scorpions amplification refractory mutation system.

a Fully detectable: full spectrum of mutation(s) could be detected correctly.

b Partly detectable: part of complex mutations could be detected.

## Discussion

A subset of patients who do not harbor *EGFR* mutations could benefit from EGFR-TKIs treatment and a pooled analysis by Lindeman et al. showed an 11% ORR of EGFR-TKIs in patients with *EGFR*-wt NSCLC assessed by various detection methods [28]. Similar to this result, our study showed that ORR of erlotinib in Group-I patients was 14.6%. By the cross recheck of *EGFR* mutation status, we found that more than half of the erlotinib responders actually harbored *EGFR* mutations and both DS and MtS methods were unable to detect a significant portion of *EGFR*-mutations.

In 2011, Naoki et al. compared the detection sensitivity of DS and PCR-invader method and reported that *EGFR* mutations were detected in 52% of the samples with PCR-invader method but only 35% of the samples by DS [16]. Similar results have been reported when DS was compared with other MtS methods [14], [29]. In the present study, 5 of 10 erlotinib responders (50.0%), who had *EGFR*-wt tumors by DS, were found to harbor *EGFR* mutations by MALDI-TOF MS. These results provided evidence that the relative low sensitivity of DS could account for some of the responses to erlotinib in patients without detectable *EGFR* mutations.

In the present study, we divided patients into DS and MtS groups depending on which methods used at initial molecular testing. As the relative low sensitivity of DS has been suspected to be a possible reason for the responses to EGFR-TKIs in patients without detectable *EGFR* mutations [14], [15], a better outcome would be expected in the group detected by DS because it could miss more *EGFR*-mutant patients. However, in the present study, we found that neither responsiveness nor survival time correlated significantly with detection methods. These results suggested that there might be also limitations in MtS methods that could potentially lead to failure to detect *EGFR* mutations in some patients.

MtS methods have higher detection sensitivity but might not be able to detect rare and unknown mutations. In contrast to DS, only a few studies have focused on the impact of the limitations of MtS methods [17], [30]. Though the exon 19 deletions and L858R account for the majority of *EGFR* mutations, patients harboring other uncommon mutations could also benefit from EGFR-TKIs therapy [31]. Yang et al. has suggested that the absence of an *EGFR* mutation, as determined by methods that only detect known mutations, should not be used as an exclusion criterion for the EGFR-TKIs therapy [17]. In the present study, 5 of 9 erlotinib responders (55.6%), who had *EGFR*-wt tumors by MtS methods, were found to possess *EGFR* mutations by DS. Moreover, analysis of an independent DS cohort showed that about 8% of *EGFR* mutations might be undetectable by MtS methods and the accuracy rates would be less than 90%. Moreover, a significant portion of these uncommon mutations is associated with disease control in response to EGFR-TKIs therapy. According to the Catalogue of Somatic Mutations in Cancer (COSMIC) (v66) database, 9.5% (524 of 5544 reported cases) of the *EGFR* mutations in lung adenocarcinoma may be undetected by modern MtS methods [32], a finding which was similar to that of our study. Our study highlighted the limitations of MtS methods.

A recently published molecular testing guideline for *EGFR* mutations suggested that laboratories are strongly encouraged to use sensitive methods that are able to detect mutations in specimens with as little as 10% cancer cells [28], However, our results indicated that both DS and MtS methods have strengths and weaknesses and could potentially miss part of *EGFR*-mutant patients. Recently, Er et al. also focused on this issue and suggested that all samples should be screened by MtS methods first and if the mutation is detected, the results could be reported directly. If no mutation can be found, the samples should be rechecked by DS. The results indicated that combination strategy as real-time PCR screening followed by DS could increase the *EGFR* mutation detection rate by 4% [30]. In regions with higher frequency of *EGFR* mutations, there could miss more *EGFR*-mutant patients as the similar false negative rates of detection methods. The cost-effectiveness should also be considered in determining which strategy is suitable for clinical settings.

In the present study, there were 9 patients, who were really *EGFR*-wt by both DS and MtS methods, achieved PR to erlotinib treatment. One possible reason for the response to erlotinib in *EGFR*-wt NSCLC is that erlotinib might target pathways related to antitumor activity other than the *EGFR* mutations because objective responses to erlotinib have been independently observed in *EGFR*-wt NSCLC patients, not only in those with adenocarcinoma but also in the squamous cell carcinoma subgroup, which usually has a low *EGFR* mutation rate [9], [33]. Previous studies have suggested potential mechanisms to explain the erlotinib activities in *EGFR*-wt lung cancers, such as *EGFR* copy numbers [34], mutations in other exons of the *EGFR* gene [35], cancerous inhibitor of protein phosphatase 2A (CIP2A) pathway [36] and VeriStrat status [37]. Further studies are needed to define the underlying mechanisms.

Recent advances in sequencing methods, such as next-generation technologies, could provide a rapid, multiplexed, ultrasensitive and high throughput detection of *EGFR* and other actionable mutations [38]. Furthermore, a recent study by Couraud et al. suggested the potential utility of using next-generation sequencing to non-invasively screen actionable mutations in plasma cell-free DNA in lung cancer patients [39]. These results may provide another aspect on future targeted molecular therapy.

In conclusion, a significant portion of the erlotinib responses in lung adenocarcinoma patients without detectable *EGFR* mutations was related to the limitations of detection methods. We further highlighted that not only DS but also MtS methods were unable to detect *EGFR* mutations in some patients. Prospective studies are needed to define the proper strategy for *EGFR* mutation testing in order to enable more patients to undergo EGFR-TKIs therapy, which should take balance between the cost-effectiveness and detection sensitivity.

## Supporting Information

Table S1 ***EGFR*** **mutations detected by PNA-LNA PCR clamp and MALDI-TOF MS.**
(PDF)

Table S2 **Demographic data of the Group-I patients.**
(PDF)

Table S3 **Demographic data of the Group-II patients.**
(PDF)

Table S4 ***EGFR*** **mutation spectrum, detectability and responses to EGFR-TKIs treatment of an independent direct sequencing cohort.**
(PDF)

## References

[1] Lee CK, Brown C, Gralla RJ, Hirsh V, Thongprasert S, et al (2013) Impact of EGFR inhibitor in non-small cell lung cancer on progression-free and overall survival: a meta-analysis. J Natl Cancer Inst 105: 595–605. 23594426 10.1093/jnci/djt072

[2] Chen G, Feng J, Zhou C, Wu YL, Liu XQ, et al (2013) Quality of life (QoL) analyses from OPTIMAL (CTONG-0802), a phase III, randomised, open-label study of first-line erlotinib versus chemotherapy in patients with advanced EGFR mutation-positive non-small-cell lung cancer (NSCLC). Ann Oncol 24: 1615–1622. 23456778 10.1093/annonc/mdt012

[3] Thongprasert S, Duffield E, Saijo N, Wu YL, Yang JC, et al (2011) Health-related quality-of-life in a randomized phase III first-line study of gefitinib versus carboplatin/paclitaxel in clinically selected patients from Asia with advanced NSCLC (IPASS). J Thorac Oncol 6: 1872–1880. 22011650 10.1097/JTO.0b013e31822adaf7

[4] Yang JC, Hirsh V, Schuler M, Yamamoto N, O'Byrne KJ, et al (2013) Symptom control and quality of life in LUX-Lung 3: a phase III study of afatinib or cisplatin/pemetrexed in patients with advanced lung adenocarcinoma with EGFR mutations. J Clin Oncol 31: 3342–3350. 23816967 10.1200/JCO.2012.46.1764

[5] Leighl NB (2012) Treatment paradigms for patients with metastatic non-small-cell lung cancer: first-, second-, and third-line. Curr Oncol 19: S52–58. 22787411 10.3747/co.19.1114 PMC3377755

[6] Reck M, Heigener DF, Mok T, Soria JC, Rabe KF (2013) Management of non-small-cell lung cancer: recent developments. Lancet 382: 709–719. 23972814 10.1016/S0140-6736(13)61502-0

[7] Chen YM, Tsai CM, Fan WC, Shih JF, Liu SH, et al (2012) Phase II randomized trial of erlotinib or vinorelbine in chemonaive, advanced, non-small cell lung cancer patients aged 70 years or older. J Thorac Oncol 7: 412–418. 22157367 10.1097/JTO.0b013e31823a39e8

[8] Kobayashi T, Koizumi T, Agatsuma T, Yasuo M, Tsushima K, et al (2012) A phase II trial of erlotinib in patients with EGFR wild-type advanced non-small-cell lung cancer. Cancer Chemother Pharmacol 69: 1241–1246. 22278730 10.1007/s00280-012-1831-0

[9] Tseng JS, Yang TY, Chen KC, Hsu KH, Chen HY, et al (2012) Retrospective study of erlotinib in patients with advanced squamous lung cancer. Lung Cancer 77: 128–133. 22420950 10.1016/j.lungcan.2012.02.012

[10] Azzoli CG, Temin S, Giaccone G (2012) 2011 Focused Update of 2009 American Society of Clinical Oncology Clinical Practice Guideline Update on Chemotherapy for Stage IV Non-Small-Cell Lung Cancer. J Oncol Pract 8: 63–66. 22548014 10.1200/JOP.2011.000374 PMC3266319

[11] Peters S, Adjei AA, Gridelli C, Reck M, Kerr K, et al (2012) Metastatic non-small-cell lung cancer (NSCLC): ESMO Clinical Practice Guidelines for diagnosis, treatment and follow-up. Ann Oncol 23 Suppl 7 vii56–64. 22997455 10.1093/annonc/mds226

[12] National Comprehensive Cancer Network (2014) The NCCN clinical practice guidelines in oncology (NCCN guidelines) for non-small cell lung cancer. Version 3.2014. http://www.nccn.org. 10.6004/jnccn.2022.0025 35545176

[13] Jazieh AR, Al Sudairy R, Abu-Shraie N, Al Suwairi W, Ferwana M, et al (2013) Erlotinib in wild type epidermal growth factor receptor non-small cell lung cancer: A systematic review. Ann Thorac Med 8: 204–208. 24250733 10.4103/1817-1737.118503 PMC3821279

[14] Horiike A, Kimura H, Nishio K, Ohyanagi F, Satoh Y, et al (2007) Detection of epidermal growth factor receptor mutation in transbronchial needle aspirates of non-small cell lung cancer. Chest 131: 1628–1634. 17565015 10.1378/chest.06-1673

[15] Yoshioka H, Hotta K, Kiura K, Takigawa N, Hayashi H, et al (2010) A phase II trial of erlotinib monotherapy in pretreated patients with advanced non-small cell lung cancer who do not possess active EGFR mutations: Okayama Lung Cancer Study Group trial 0705. J Thorac Oncol 5: 99–104. 19898258 10.1097/JTO.0b013e3181c20063

[16] Naoki K, Soejima K, Okamoto H, Hamamoto J, Hida N, et al (2011) The PCR-invader method (structure-specific 5′ nuclease-based method), a sensitive method for detecting EGFR gene mutations in lung cancer specimens; comparison with direct sequencing. Int J Clin Oncol 16: 335–344. 21311943 10.1007/s10147-011-0187-5

[17] Yang TY, Tsai CR, Chen KC, Hsu KH, Lee HM, et al (2011) Good response to gefitinib in a lung adenocarcinoma harboring a heterozygous complex mutation of L833V and H835L in epidermal growth factor receptor gene. J Clin Oncol 29: e468–469. 21422421 10.1200/JCO.2010.33.5802

[18] Chiu CH, Ho HL, Chiang CL, Lin SF, Ma HH, et al (2014) Clinical characteristics and treatment outcomes of lung adenocarcinomas with discrepant EGFR mutation testing results derived from PCR-direct sequencing and real-time PCR-based assays. J Thorac Oncol 9: 91–96. 24346097 10.1097/JTO.0000000000000041

[19] Zhou Q, Zhang XC, Chen ZH, Yin XL, Yang JJ, et al (2011) Relative abundance of EGFR mutations predicts benefit from gefitinib treatment for advanced non-small-cell lung cancer. J Clin Oncol 29: 3316–3321. 21788562 10.1200/JCO.2010.33.3757

[20] Edge SB, Byrd DR, Compton CC, Fritz AG, Greene FL, eds. AJCC Cancer Staging Handbook. 7th ed. New York: Springer; 2009.

[21] Eisenhauer EA, Therasse P, Bogaerts J, Schwartz LH, Sargent D, et al (2009) New response evaluation criteria in solid tumours: revised RECIST guideline (version 1.1). Eur J Cancer 45: 228–247. 19097774 10.1016/j.ejca.2008.10.026

[22] Chou TY, Chiu CH, Li LH, Hsiao CY, Tzen CY, et al (2005) Mutation in the tyrosine kinase domain of epidermal growth factor receptor is a predictive and prognostic factor for gefitinib treatment in patients with non-small cell lung cancer. Clin Cancer Res 11: 3750–3757. 15897572 10.1158/1078-0432.CCR-04-1981

[23] Newton CR, Graham A, Heptinstall LE, Powell SJ, Summers C, et al (1989) Analysis of any point mutation in DNA. The amplification refractory mutation system (ARMS). Nucleic Acids Res 17: 2503–2516. 2785681 10.1093/nar/17.7.2503 PMC317639

[24] Su KY, Chen HY, Li KC, Kuo ML, Yang JC, et al (2012) Pretreatment epidermal growth factor receptor (EGFR) T790M mutation predicts shorter EGFR tyrosine kinase inhibitor response duration in patients with non-small-cell lung cancer. J Clin Oncol 30: 433–440. 22215752 10.1200/JCO.2011.38.3224

[25] EGFR RGQ PCR Kit Product Details. Available: http://www.qiagen.com/products/catalog/assay-technologies/complete-assay-kits/personalized-healthcare/egfr-rgq-pcr-kit - productdetails/. Accessed 2014 July 29.

[26] Kimura H, Ohira T, Uchida O, Matsubayashi J, Shimizu S, et al (2014) Analytical performance of the cobas EGFR mutation assay for Japanese non-small-cell lung cancer. Lung Cancer 83: 329–333. 24439568 10.1016/j.lungcan.2013.12.012

[27] Yeh P, Chen H, Andrews J, Naser R, Pao W, et al (2013) DNA-Mutation Inventory to Refine and Enhance Cancer Treatment (DIRECT): a catalog of clinically relevant cancer mutations to enable genome-directed anticancer therapy. Clin Cancer Res 19: 1894–1901. 23344264 10.1158/1078-0432.CCR-12-1894 PMC4121886

[28] Lindeman NI, Cagle PT, Beasley MB, Chitale DA, Dacic S, et al (2013) Molecular testing guideline for selection of lung cancer patients for EGFR and ALK tyrosine kinase inhibitors: guideline from the College of American Pathologists, International Association for the Study of Lung Cancer, and Association for Molecular Pathology. J Thorac Oncol 8: 823–859. 23552377 10.1097/JTO.0b013e318290868f PMC4159960

[29] Kim HJ, Lee KY, Kim YC, Kim KS, Lee SY, et al (2012) Detection and comparison of peptide nucleic acid-mediated real-time polymerase chain reaction clamping and direct gene sequencing for epidermal growth factor receptor mutations in patients with non-small cell lung cancer. Lung Cancer 75: 321–325. 21930325 10.1016/j.lungcan.2011.08.005

[30] Er TK, Lin CW, Liu TC, Chen CC, Wang LH, et al. (2014) Increase EGFR Mutations Detection Rate in Lung Adenocarcinoma by Real-Time PCR Screening Followed by Direct Sequencing. Diagn Mol Pathol. 10.1097/PDM.0000000000000037 25961746

[31] Wu JY, Yu CJ, Chang YC, Yang CH, Shih JY, et al (2011) Effectiveness of tyrosine kinase inhibitors on “uncommon” epidermal growth factor receptor mutations of unknown clinical significance in non-small cell lung cancer. Clin Cancer Res 17: 3812–3821. 21531810 10.1158/1078-0432.CCR-10-3408

[32] Catalogue of Somatic Mutations in Cancer. [http://cancer.sanger.ac.uk/cancergenome/projects/cosmic/.

[33] Chiang CL, Tsai CM, Chou TY, Chen YM, Lai SL, et al (2013) Erlotinib in patients with advanced lung squamous cell carcinoma. Cancer Chemother Pharmacol 71: 203–208. 23053274 10.1007/s00280-012-1997-5

[34] Wang F, Fu S, Shao Q, Zhou YB, Zhang X, et al (2013) High EGFR copy number predicts benefits from tyrosine kinase inhibitor treatment for non-small cell lung cancer patients with wild-type EGFR. J Transl Med 11: 90. 23557218 10.1186/1479-5876-11-90 PMC3635875

[35] Cho J, Pastorino S, Zeng Q, Xu X, Johnson W, et al (2011) Glioblastoma-derived epidermal growth factor receptor carboxyl-terminal deletion mutants are transforming and are sensitive to EGFR-directed therapies. Cancer Res 71: 7587–7596. 22001862 10.1158/0008-5472.CAN-11-0821 PMC3242822

[36] Wang CY, Chao TT, Chang FY, Chen YL, Tsai YT, et al (2014) CIP2A mediates erlotinib-induced apoptosis in non-small cell lung cancer cells without EGFR mutation. Lung Cancer 85: 152–160. 24954871 10.1016/j.lungcan.2014.05.024

[37] Gregorc V, Novello S, Lazzari C, Barni S, Aieta M, et al (2014) Predictive value of a proteomic signature in patients with non-small-cell lung cancer treated with second-line erlotinib or chemotherapy (PROSE): a biomarker-stratified, randomised phase 3 trial. Lancet Oncol 15: 713–721. 24831979 10.1016/S1470-2045(14)70162-7

[38] Wu K, Huang RS, House L, Cho WC (2013) Next-generation sequencing for lung cancer. Future Oncol 9: 1323–1336. 23980680 10.2217/fon.13.102

[39] Couraud S, Vaca Paniagua F, Villar S, Oliver J, Schuster T, et al. (2014) Non-invasive diagnosis of actionable mutations by deep sequencing of circulating-free DNA in non-small cell lung cancer: Findings from BioCAST/IFCT-1002. Clin Cancer Res. 10.1158/1078-0432.CCR-13-3063 25013125

