# Supplementary material for: Impact of EGFR Mutation Detection Methods on the Efficacy of Erlotinib in Patients with Advanced EGFR-Wild Type Lung Adenocarcinoma
Source: PLoS One. 2014 Sep 12;9(9):e107160. doi: 10.1371/journal.pone.0107160 (PMC4162576; doi:10.1371/journal.pone.0107160)
Supplement: Table S1 — EGFR mutations detected by PNA-LNA PCR clamp and MALDI-TOF MS. (PDF) [file pone.0107160.s001.pdf]

Table S1. *EGFR* mutations detected by PNA-LNA PCR clamp and MALDI-TOF MS

Exon	Mutation types (nucleic acid sequence)
**PNA-LNA PCR clamp**	
18	G719A (2156G>C), G719C (2155G>T), G719S (2155G>A)
19	Del E746_A750 (2235_2249del15, 2236_2250del15),
	Del L747_T751 (2239_2253del15),
	Del L747_P753>S (2240_2257del18, 2239_2259>AGC)
20	T790M (2369C>T)
21	L858R (2573T>G), L861Q (2582T>A)
**MALDI-TOF MS**	
18	E709A (2126A>C), E709G (2126A>G), E709V (2126A>T), G719A (2156G>C), G719C (2155G>T), G719N (2156G>A), G719S (2155G>A)
19	Del E746_A750 (2235_2249del15, 2236_2250del15),
	Del E746_T751 (2236_2253del18),
	Del E746_T751>A (2237_2251del15),
	Del E746-T751>I (2235_2252>AAT),
	Del E746_S752>A (2237_2254del18),
	Del E746_S752>D (2238_2255del18),
	Del E746_S752>V (2237_2255>T),
	Del L747_E749 (2239_2247del9),
	Del L747_A750>P (2238_2248>GC, 2239_2248TTAAGAGAAG>C),
	Del L747_T751 (2239_2253del15, 2240_2254del15),
	Del L747_T751>P (2239_2251>C),
	Del L747_T751>Q (2238_2252>GCA),
	Del L747_T751>S (2240_2251del12),
	Del L747_S752 (2239_2256del18),
	Del L747_P753>Q (2239_2258>CA),
	Del L747_P753>S (2240_2257del18)
20	S768I (2303G>T), T790M (2369C>T)
21	L858Q (2573T>A), L858R (2573T>G), L861Q (2582T>A)

EGFR, epidermal growth factor receptor; PNA-LNA PCR, protein nucleic acid-locked nucleic acid polymerase chain reaction; MALDI-TOF MS, matrix-assisted laser desorption ionization-time of flight mass spectrometry.
