# Supplementary material for: Impact of EGFR Mutation Detection Methods on the Efficacy of Erlotinib in Patients with Advanced EGFR-Wild Type Lung Adenocarcinoma
Source: PLoS One. 2014 Sep 12;9(9):e107160. doi: 10.1371/journal.pone.0107160 (PMC4162576; doi:10.1371/journal.pone.0107160)
Supplement: Table S2 — Demographic data of the Group-I patients. (PDF) [file pone.0107160.s002.pdf]

Table S2. Demographic data of the Group-I patients

Characteristics	N = 261
Age (years), median (range)	62 (25-90)
Gender, n (%)	
Male	162 (62.1)
Female	99 (37.9)
Smoking status, n (%)	
Non-smoker	138 (52.9)
Former-smoker	56 (21.5)
Current-smoker	67 (25.7)
ECOG PS, n (%)	
0-1	174 (66.7)
2 or more	87 (33.3)
Stage, n (%)	
IIIb	20 (7.7)
IV	241 (92.3)
Prior chemotherapy, n (%)	
0	34 (13.0)
1	133 (51.0)
2	58 (22.2)
3 or more	36 (13.8)
EGFR detection methods, n (%)	
Direct sequencing	191 (73.2)
PNA-LNA PCR Clamping	42 (16.1)
MALDI-TOF MS	14 (5.4)
ARMS	14 (5.4)

ECOG PS, Eastern Cooperative Oncology Group performance status; EGFR, epidermal growth factor receptor; PNA-LNA, protein nucleic acid-locked nucleic acid; MALDI-TOF MS, matrix-assisted laser desorption ionization-time of flight mass spectrometry; ARMS, Scorpions amplification refractory mutation system.
