# Supplementary material for: Impact of EGFR Mutation Detection Methods on the Efficacy of Erlotinib in Patients with Advanced EGFR-Wild Type Lung Adenocarcinoma
Source: PLoS One. 2014 Sep 12;9(9):e107160. doi: 10.1371/journal.pone.0107160 (PMC4162576; doi:10.1371/journal.pone.0107160)
Supplement: Table S3 — Demographic data of the Group-II patients. (PDF) [file pone.0107160.s003.pdf]

Table S3. Demographic data of the Group-II patients

Characteristics	N = 996
Age (years), median (range)	64 (25-98)
Gender, n (%)	
Male	502 (50.4)
Female	494 (49.6)
Smoking status, n (%)	
Non-smoker	664 (66.7)
Former-smoker	85 (8.5)
Current-smoker	236 (23.7)
N/A	11 (1.1)
Stage , n (%)	
I	118 (11.8)
II	49 (4.9)
IIIa	78 (7.8)
IIIb	66 (6.6)
IV	685 (68.8)

N/A, not applicable.
