# Supplementary material for: Impact of EGFR Mutation Detection Methods on the Efficacy of Erlotinib in Patients with Advanced EGFR-Wild Type Lung Adenocarcinoma
Source: PLoS One. 2014 Sep 12;9(9):e107160. doi: 10.1371/journal.pone.0107160 (PMC4162576; doi:10.1371/journal.pone.0107160)
Supplement: Table S4 — EGFR mutation spectrum, detectability and responses to EGFR-TKIs treatment of an independent direct sequencing cohort. (PDF) [file pone.0107160.s004.pdf]

Table S4. *EGFR* mutation spectrum, detectability and responses to EGFR-TKIs treatment of an independent direct sequencing cohort

			Detectability of MtS methods*			DC by EGFR-TKIs**	
Mutation type	Exon	Pt No.	MS	ARMS	Cobas	DIRECT[a]	TH/CH[b]
Del E709_T710	18	1					
E709A	18	1	●			△	
E709K	18	1				△	
E709V	18	1	●				▲
E711_F712>N	18	1					
G719A	18	4	●	●	●	▲	▲
G719C	18	1	●	●	●	▲	▲
G719S	18	2	●	●	●	▲	▲
E709K/G719A	18	1	○	○	○	▲	
E709V/G719A	18	1	●	○	○	△	
E709A/G719S	18	2	●	○	○	△	▲
G719A/L747S	18, 19	1	○	○	○	△	
G719A/R776H	18, 20	1	○	○	○	△	▲
G719A/T790M	18, 20	1	●	●	●	△	
G719C/S768I	18, 20	1	●	●	●	▲	▲
G719S/S768I	18, 20	2	●	●	●	△	
V689L/L858R	18, 21	1	○	○	○	▲	
Q701L/L858R	18, 21	2	○	○	○	△	▲
E709G/L858R	18, 21	2	●	○	○	▲	▲
E709K/L858R	18, 21	3	○	○	○	▲	▲
E709V/L858R	18, 21	1	●	○	○	△	▲
K716Q/G719A/L861Q	18, 21	1	○	○	○	△	▲
G719C/L861Q	18, 21	1	●	●	○	△	
P743S	19	1					▲
I744V/Del E746-A750	19	1	○	○	○	△	▲
I744_K745>KIPVAI	19	1					
Del K745_A750	19	1				▲	▲
Del E746_A750	19	164	●	●	●	▲	▲

Del E746_A750>VP	19	1					▲
E746G/L747P	19	1				△	
Del E746_T751	19	1	●	●	●	▲	▲
Del E746_T751>A	19	6	●	●	●	▲	▲
Del E746_T751>L	19	1					▲
Del E746_T751>V	19	3			●	▲	
Del E746_T751>VA	19	2			●		▲
Del E746_T751>VP	19	1					▲
Del E746_T751/Del P753	19	1	○	○	○	△	▲
Del E746_S752>V	19	5	●	●	●	▲	▲
Del L747_E749	19	1	●	●	●	▲	
Del L747_E749>C	19	1					
Del L747_E749/K754E	19	1	○	○	○	△	
Del L747_A750>P	19	13	●	●	●	▲	▲
Del L747_T751	19	19	●	●	●	▲	▲
Del L747_T751>P	19	5	●	●	●	▲	▲
Del L747-T751>PI	19	1					
Del L747_S752	19	3	●	●	●	▲	▲
Del L747_S752>Q	19	1			●	▲	▲
Del L747_P753>S	19	14	●	●	●	▲	▲
Del L747_P753>SK	19	1					
Del A750_I759>SS	19	1					▲
Del T751_I759>N	19	1					▲
Del T751_I759>S	19	1					▲
Del S752_I759	19	1			●	▲	▲
Del L747_P753>S/V834L	19, 21	1	○	○	○	△	▲
L747S/L858R	19, 21	2	○	○	○	△	▲
D761_E762>EAFQ	20	1					
M766_A767>PHVC	20	1					
A767_V769 dup	20	4					▲
S768_V769>NPH	20	1					
S768_D770 dup	20	1					

V769_D770>ASV	20	1		●	●		
D770_N771>SVD	20	3			●		
Del D770_N771>PPH	20	1					
H773_V774>H	20	1		●	●		
D807N	20	1					
D761Y/L858R	20, 21	1	○	○	○	▲	
S768I/L858R	20, 21	3	●	●	●	▲	▲
V769A/L858R	20, 21	1	○	○	○	△	▲
V769M/L858R	20, 21	1	○	○	○	△	▲
R776H/L858R	20, 21	2	○	○	○	▲	▲
T790M/L858R	20, 21	5	●	●	●	△	▲
L833F/L861R	21	1				△	▲
L833V/H835L	21	3				△	▲
L833V/L858R	21	2	○	○	○	▲	▲
L858R	21	251	●	●	●	▲	▲
L858M/L858R	21	1	○	○	○	△	
L858R/V834L	21	4	○	○	○	▲	▲
L858R/V843I	21	1	○	○	○	▲	▲
L861Q	21	15	●	●		▲	▲
L861Q/E866Q	21	1	○	○		△	▲
L861R	21	1				▲	▲

DC, disease control, MtS, mutant type-specific sensitive methods; EGFR-TKIs, epidermal growth factor receptor-tyrosine kinase inhibitors; MS, matrix-assisted laser desorption ionization-time of flight mass spectrometry; ARMS, Scorpions amplification refractory mutation system.

[a]DIRECT: mutations associated with disease control to EGFR-TKIs by DNA-Mutation Inventory to Refine and Enhance Cancer Treatment (DIRECT) database (Clin Cancer Res 2013; 19:1894-1901).

[b]TH/CH: mutations associated with disease control to EGFR-TKIs by cases with treatment history at Taichung Veterans General Hospital and Chang Gung Memorial Hospital.

[*]Detectability: ●Fully detectable; ○Partly detectable.

[**]Disease control (DC) by EGFR-TKIs: ▲Matched with mutation(s) associated with disease control in DIRECT database or cases with disease control in response to EGFR-TKIs therapy at our facilities; △ Matched with part of mutation(s) associated with disease control in DIRECT database.
